# Supplementary material for: Effect of Landscape Composition and Invasive Plants on Pollination Networks of Smallholder Orchards in Northeastern Thailand
Source: Plants (Basel). 2022 Jul 29;11(15):1976. doi: 10.3390/plants11151976 (PMC9370323; doi:10.3390/plants11151976)
Supplement: Supplementary file 1 [file plants-11-01976-s001.zip › plants-1834977-supplementary.pdf]

Table S1. The list of plant and pollinator species in the network of all study sites, non-invaded network, and invaded network.

Network	Plant species	
The overall network (Figure 2a)	P1 *Ixora chinensis* Lamk. (Rubiaceae)	P35 *Gomphrena globosa* L. (Amaranthaceae)
	P2 *Nephelium lappaceum* L. (Sapindaceae)	P36 *Zinnia violacea* Cav. (Asteraceae)
	P3 *Sesbania grandiflora* (L.) Poir. (Fabaceae)	P37 *Asystasia gangetica* (L.) T. Anderson subsp. gangetica (Acanthaceae)
	P4 *Wrightia religiosa* (Teijsm. & Binn.) Benth. ex (Apocynaceae)	P38 *Bidens pilosa* L. (Asteraceae)
	P5 *Ocimum basilicum* L. (Lamiaceae)	P39 *Syzygium antisepticum* (Blume) Merr. & L. M. Perry (Myrtaceae)
	P6 *Mimosa pudica* L. (Fabaceae)	P40 *Cleome gynandra* L. (Cleomaceae)
	P7 *Scoparia dulcis* L. (Plantaginaceae)	P41 *Cnidoscolus aconitifolius* (Mill.) I. M. Johnst. (Euphorbiaceae)
	P8 *Sandoricum koetjape* (Burm. f.) Merr. (Meliaceae)	P42 *Brassica juncea* (L.) Czern. (Brassicaceae)
	P9 *Ocimum tenuiflorum* L. (Lamiaceae)	P43 *Eryngium foetidum* L. (Apiaceae)
	P10 *Passiflora laurifolia* L. (Passifloraceae)	P44 *Anethum graveolens* L. (Apiaceae)
	P11 *Coffea arabica* L. (Rubiaceae)	P45 *Psidium guajava* L. (Myrtaceae)
	P12 *Streblus asper* Lour. (Moraceae)	P46 *Capsicum annuum* L. (Solanaceae)
	P13 *Justicia gendarussa* Burm. f. (Acanthaceae)	P47 *Phytolacca americana* L. (Phytolacaceae)
	P14 *Zea mays* L. (Poaceae)	P48 *Benincasa hispida* (Thunb.) Cogn. (Cucurbitaceae)
	P15 *Cosmos bipinnatus* Cav. (Asteraceae)	P49 *Solanum sp.* (Solanaceae)
	P16 *Ipomoea obscura* (L.) Ker Gawl. (Convolvulaceae)	P50 *Solanum lycopersicum* L. (Solanaceae)
	P17 *Vallaris glabra* (L.) Kuntze (Apocynaceae)	P51 *Solanum incanum* L. (Solanaceae)
	P18 *Calotropis gigantea* (L.) Dryand. (Apocynaceae)	P52 *Solanum torvum* Sw. (Solanaceae)
	P19 *Tagetes erecta* L. (Asteraceae)	P53 *Solanum melongena* L. (Solanaceae)
	P20 *Glossocardia bidens* (Retz.) Veldkamp (Asteraceae)	P54 *Averrhoa carambola* L. (Oxalidaceae)
	P21 *Allium fistulosum* L. (Amaryllidaceae)	P55 *Pithecellobium dulce* (Roxb.) Benth. (Fabaceae)
	P22 *Hygrophila erecta* (Burm. f.) Hochr. (Acanthaceae)	P56 *Citrus aurantifolia* (Christm.) Swingle (Rutaceae)
	P23 *Muntingia calabura* L. (Muntingiaceae)	P57 *Bouae burmanica* Griff. (Anacardiaceae)
	P24 *Averrhoa bilimbi* L. (Oxalidaceae)	P58 *Cocos nucifera* L. (Arecaceae)
	P25 *Coccinia grandis* (L.) Voigt (Cucurbitaceae)	P59 *Mangifera indica* Linn. (Anacardiaceae)
	P26 *Tridax procumbens* (L.) L. (Asteraceae)	P60 *Carissa carandas* L. (Apocynaceae)
	P27 *Lablab purpureus* (L.) Sweet. (Fabaceae)	P61 *Momordica charantia* L. (Cucurbitaceae)
	P28 *Centrosema pubescens* Benth. (Fabaceae)	P62 *Moringa oleifera* Lam. (Moringaceae)
	P29 *Rhinacanthus nasutus* (L.) Kurz. (Acanthaceae)	P63 *Carica papaya* L. (Caricaceae)
	P30 *Tecoma stans* (L.) Kunth (Bignoniaceae)	P64 *Morinda citrifolia* L. (Rubiaceae)
	P31 *Punica granatum* L. granatum (Lythraceae)	P65 *Nicotiana tabacum* L. (Solanaceae)
	P32 *Nelumbo nucifera* Gaertn. (Nelumbonaceae)	P66 *Dimocarpus longan* Lour. (Sapindaceae)
	P33 *Nelumbo* sp.1 (Nelumbonaceae)	P67 *Litchi chinensis* Sonn. (Sapindaceae)
	P34 *Nelumbo* sp.2 (Nelumbonaceae)	P68 *Citrus maxima* (Burm.f.) Merr. (Rutaceae)
**Network**	**Plant species**	

	P69 *Telosma cordata* (Burm. f.) Merr. (Apocynaceae)		P72 *Heliotropium indicum* L. (Boraginaceae)	
	P70 *Azadirachta indica* A. Juss. (Meliaceae)		P73 *Areca catechu* L. (Arecaceae)	
	P71 *Praxelis clematidae* RM King & H. Rob (Asteraceae)			
	**Pollinator species**			
	A1 *Apis cerana*	A34 *Xylocopa sp.1*	A67 *Vespid sp.7*	A100 *Tephritidae sp.1*
	A2 *Apis florea*	A35 *Xylocopa sp.2*	A68 *Formicid sp.1*	A101 *Tephritidae sp.2*
	A3 *Amegilla sp.1*	A36 *Xylocopa aestuans*	A69 *Formicid sp.2*	A102 *Dolichopodid sp.2*
	A4 *Amegilla sp.2*	A37 *Xylocopa sp.4*	A70 *Anoplolepis gracilipes*	A103 *Musca sp.*
	A5 *Apidae sp.1*	A38 *Xylocopa sp.5*	A71 *Formicid sp.6*	A104 *Lixophaga sp.*
	A6 *Apidae sp.2*	A39 *Amegilla sp.3*	A72 *Formicid sp.7*	A105 *Syritta sp.*
	A7 *Halictid sp.2*	A40 *Amegilla sp.4*	A73 *Formicid sp.8*	A106 *Tephritidae sp.*
	A8 *Andrena sp.2*	A41 *Megachile disjuncta*	A74 *Formicid sp.9*	A107 *Mesembrius bengalensis*
	A9 *Andrena sp.3*	A42 *Euaspis sp.*	A75 *Formicid sp.10*	A108 *Dideopsis aegrota*
	A10 *Halictid sp.3*	A43 *Apidae sp.4*	A76 *Formicid sp.5*	A109 *Syrphidae sp.2*
	A11 *Ceratina sp.*	A44 *Megascolia sp.1*	A77 *Formicid sp.3*	A110 *Episyrphus sp.2*
	A12 *Halictid sp.4*	A45 *Megascolia sp.2*	A78 *Formicid sp.11*	A111 *Syrphidae sp.3*
	A13 *Symmorphus sp.*	A46 *Megascolia sp.4*	A79 *Formicid sp.12*	A112 Small-yellow fly
	A14 *Apidae sp.5*	A47 *Megascolia sp.5*	A80 *Formicid sp.13*	A113 *Sarcophagidae sp.5*
	A15 *Halictid sp.7*	A48 *Vespid sp.5*	A81 Banded fly	A114 *Sarcophagidae sp.7*
	A16 *Augochlora sp.*	A49 *Vespid sp.12*	A82 *Stratiomyid sp.2*	A115 *Sarcophagidae sp.6*
	A17 *Andrena sp.1*	A50 *Eumeninae sp.1*	A83 *Sarcophagidae sp.4*	A116 *Stratiomyid sp.7*
	A18 *Tetragonilla sp.2*	A51 *Vespid sp.4*	A84 *Calliphoridae sp.1*	A117 *Stomorhina sp.*
	A19 Tetragonilla sp.3	A52 *Eumeninae sp.2*	A85 *Helophilu sp.*	A118 *Episyrphus sp.1*
	A20 *Tetragonilla sp.4*	A53 *Polistinae sp.1*	A86 *Stratiomyid sp.9*	A119 *Stratiomyid sp.8*
	A21 *Tetragonilla sp.5*	A54 *Polistinae sp.2*	A87 *Stratiomyid sp.3*	A120 *Stratiomyid sp.5*
	A22 *Halictid sp.5*	A55 *Vespid sp.1*	A88 *Stratiomyid sp.6*	A121 Metallic-black fly
	A23 *Halictid sp.6*	A56 *Vespid sp.2*	A89 *Dolichopodid sp.1*	A122 Black-white fly
	A24 *Megachile sp.1*	A57 *Phimenes sp.*	A90 *Stratiomyid sp.1*	A123 White fly
	A25 *Tetragonula sp.1*	A58 *Eumeninae sp.3*	A91 *Lucilia illustris*	A124 *Stratiomyid sp.4*
	A26 *Tetragonula sp.2*	A59 *Vespid sp.3*	A92 *Sarcophagidae sp.1*	A125 *Syrphidae sp.1*
	A27 *Halictid sp.1*	A60 *Vespid sp.8*	A93 *Sarcophagidae sp.2*	A126 *Drosophila sp.2*
	A28 *Lophotrigona sp.*	A61 *Vespid sp.9*	A94 *Sarcophaga sp.*	A127 *Drosophila sp.1*
	A29 *Tetragonilla sp.1*	A62 *Vespid sp.10*	A95 *Muscid sp.1*	A128 *Papilio demolion*
	A30 *Apidae sp.3*	A63 *Vespid sp.11*	A96 *Calliphoridae sp.2*	A129 *Catopsilia pomona*
	A31 *Megachile sp.2*	A64 *Campsomerinae sp.*	A97 *Muscid sp.2*	A130 *Catopsilia scylla*
	A32 *Megachile sp.3*	A65 *Pompilidae sp.1*	A98 *Muscid sp.3*	A131 *Papilio demoleus*
	A33 *Xylocopa sp.3*	A66 *Eumeninae sp.5*	A99 *Muscid sp.4*	A132 *Pseudozizeeria maha*

**Network**	**Pollinator species**

	A133 *Everes huegelii*	A145 *Papilio palytes*	A157 *Euploea sp.*	A169 *Coccinella sp.2*
	A134 *Tongeia sp.1*	A146 *Castalius rosimon*	A158 *Hypolimnas bolina*	A170 *Coreidae sp.*
	A135 *Zizina otis*	A147 *Pachliopta aristolochiae*	A159 *Junonia iphita*	A171 *Pentatomidae sp.3*
	A136 *Tongeia sp.2*	A148 *Neptis hylas*	A160 *Cirrochroa tyche*	A172 *Pentatomidae sp.2*
	A137 *Euthalia aconthea*	A149 *Melanitis leda*	A161 *Coccinella sp.1*	A173 *Pentatomidae sp.1*
	A138 *Acraea violae*	A150 *Papilio sp.*	A162 *Mordellistina sp.1*	A174 *Spilostethus sp.*
	A139 *Amata sperbius*	A151 *Leptotes plinius*	A163 *Glycyphana sp.2*	A175 *Eocanthecona furcellata*
	A140 *Danaus chrysippus*	A152 *Euploea core*	A164 *Mordellistina sp.2*	A176 *Megacopta sp.*
	A141 *Telicota colon*	A153 *Potanthus confucius*	A165 *Coccinella sp.3*	A177 Dragonfly (Libellulidae)
	A142 *Papilio memnon*	A154 *Acraea sp.*	A166 *Cheilomenes sexmaculata*	
	A143 *Erionota torus*	A155 *Euripus sp.*	A167 *Coccinella trasversalis*	
	A144 *Eurema hecabe*	A156 *Elymnias malelas*	A168 *Glycyphana sp.1*	
**Network**	**Plant species**			
Non-invaded network (Figure 2b)	P1 *Sesbania grandiflora* (L.) Poir. (Fabaceae)		P20 *Psidium guajava* L. (Myrtaceae)	
	P2 *Wrightia religiosa* (Teijsm. & Binn.) Benth. ex Kurz (Apocynaceae)		P21 *Phytolacca americana* L. (Phytolacaceae)	
	P3 *Scoparia dulcis* L. (Plantaginaceae)		P22 *Solanum incanum* L. (Solanaceae)	
	P4 *Ocimum tenuiflorum* L. (Lamiaceae)		P23 *Solanum torvum* Sw. (Solanaceae)	
	P5 *Passiflora laurifolia* L. (Passifloraceae)		P24 *Solanum melongena* L. (Solanaceae)	
	P6 *Cosmos bipinnatus* Cav. (Asteraceae)		P25 *Pithecellobium dulce* (Roxb.) Benth. (Fabaceae)	
	P7 *Ipomoea obscura* (L.) Ker Gawl. (Convolvulaceae)		P26 *Citrus aurantifolia* (Christm.) Swingle (Rutaceae)	
	P8 *Vallaris glabra* (L.) Kuntze (Apocynaceae)		P27 *Bouae burmanica* Griff. (Anacardiaceae)	
	P9 *Allium fistulosum* L. (Amaryllidaceae)		P28 *Cocos nucifera* L. (Arecaceae)	
	P10 *Averrhoa bilimbi* L. (Oxalidaceae)		P29 *Mangifera indica* L. (Anacardiaceae)	
	P11 *Coccinia grandis* (L.) Voigt (Cucurbitaceae)		P30 *Carissa carandas* L. (Apocynaceae)	
	P12 *Lablab purpureus* (L.) Sweet. (Fabaceae)		P31 *Momordica charantia* L. (Cucurbitaceae)	
	P13 *Tecoma stans* (L.) Kunth (Bignoniaceae)		P32 *Moringa oleifera* Lam. (Moringaceae)	
	P14 *Punica granatum* L. granatum (Lythraceae)		P33 *Carica papaya* L. (Caricaceae)	
	P15 *Nelumbo nucifera* Gaertn. (Nelumbonaceae)		P34 *Morinda citrifolia* L. (Rubiaceae)	
	P16 *Asystasia gangetica* (L.) T. Anderson subsp. gangetica (Acanthaceae)		P35 *Dimocarpus longan* Lour. (Sapindaceae)	
	P17 *Cleome gynandra* L. (Cleomaceae)		P36 Citrus maxima (Burm.f.) Merr. (Rutaceae)	
	P18 *Cnidoscolus aconitifolius* (Mill.) I. M. Johnst. (Euphorbiaceae)		P37 *Heliotropium indicum* L. (Boraginaceae)	
	P19 *Anethum graveolens* L. (Apiaceae)		P38 *Areca catechu* L. (Arecaceae)	
	**Pollinator species**			
	A1 *Apis cerana*	A5 *Vespid sp.6*	A9 *Lophotrigona sp.*	A13 *Halictid sp.3*
	A2 *Apis florea*	A6 *Xylocopa sp.1*	A10 *Tetragonilla sp.1*	A14 *Halictid sp.3*
	A3 *Amegilla sp.1*	A7 *Xylocopa sp.2*	A11 *Halictid sp.2*	A15 *Ceratina sp.*
	A4 *Apidae sp.1*	A8 *Xylocopa aestuans*	A12 *Andrena sp.2*	A16 *Halictid sp.4*
**Network**	**Pollinator species**			

	A17 *Symmorphus sp.*	A42 *Vespid sp.12*	A67 *Stratiomyid sp.1*	A92 *Catopsilia pomona*
	A18 *Andrena sp.1*	A43 *Vespid sp.8*	A68 *Lucilia illustris*	A93 *Catopsilia scylla*
	A19 *Megachile disjuncta*	A44 *Vespid sp.9*	A69 *Sarcophagidae sp.1*	A94 *Papilio demoleus*
	A20 *Euaspis sp.*	A45 *Vespid sp.1*	A70 *Sarcophagidae sp.2*	A95 *Pseudozizeeria maha*
	A21 *Xylocopa sp.4*	A46 *Vespid sp.11*	A71 *Sarcophaga sp.*	A96 *Everes huegelii*
	A22 *Xylocopa sp.5*	A47 *Vespa tropica*	A72 *Tephritidae sp.1*	A97 *Tongeia sp.1*
	A23 *Megachile sp.2*	A48 *Apidae sp.2*	A73 *Tephritidae sp.2*	A98 *Tongeia sp.2*
	A24 *Megachile sp.3*	A49 *Campsomerinae sp.*	A74 *Musca sp.*	A99 *Euthalia aconthea*
	A25 *Megascolia sp.2*	A50 *Formicid sp.4*	A75 *Lixophaga sp.*	A100 *Telicota colon*
	A26 *Megascolia sp.4*	A51 *Formicid sp.6*	A76 *Syritta sp.*	A101 *Melanitis leda*
	A27 *Amegilla sp.3*	A52 *Formicid sp.7*	A77 *Helophilu sp.*	A102 *Leptotes plinius*
	A28 *Amegilla sp.4*	A53 *Formicid sp.8*	A78 *Mesembrius bengalensis*	A103 *Acraea sp.*
	A29 *Halictid sp.4*	A54 *Formicid sp.9*	A79 *Dideopsis aegrota*	A104 *Euripus sp.*
	A30 *Tetragonula sp.1*	A55 *Formicid sp.1*	A80 *Syrphidae sp.1*	A105 *Euploea sp.*
	A31 *Tetragonula sp.2*	A56 *Formicid sp.3*	A81 Metallic-black fly	A106 *Hypolimnas bolina*
	A32 *Pompilidae sp.1*	A57 *Formicid sp.12*	A82 Black-white fly	A107 *Junonia iphita*
	A33 *Polistinae sp.2*	A58 *Stratiomyid sp.2*	A83 White fly	A108 *Cirrochroa tyche*
	A34 *Eumeninae sp.1*	A59 *Stomorhina sp.*	A84 *Muscid sp.4*	A109 *Mordellistina sp.2*
	A35 *Eumeninae sp.2*	A60 Small-yellow fly	A85 *Drosophila sp.1*	A110 *Glycyphana sp.1*
	A36 *Polistinae sp.1*	A61 *Sarcophagidae sp.5*	A86 *Amata sperbius*	A111 *Coreidae sp.*
	A37 *Phimenes sp.*	A62 *Stomorhina sp.*	A87 *Erionota torus*	A112 *Coccinella trasversalis*
	A38 *Eumeninae sp.3*	A63 *Episyrphus sp.1*	A88 *Papilio demolion*	A113 *Pentatomidae sp.1*
	A39 *Formicid sp.1*	A64 *Helophilu sp.*	A89 *Papilio palytes*	A114 *Megacopta sp.*
	A40 *Formicid sp.2*	A65 *Stratiomyid sp.3*	A90 *Pachliopta aristolochiae*	A115 Spider
	A41 *Apidae sp.3*	A66 *Dolichopodid sp.1*	A91 *Papilio memnon*	
**Network**	**Plant species**			
Invaded network (Figure 2c)	P1 *Nephelium lappaceum* L. (Sapindaceae)		P12 *Averrhoa carambola* L. (Oxalidaceae)	
	P2 *Mimosa pudica* L. (Fabaceae)		P13 *Azadirachta indica* A. Juss. (Meliaceae)	
	P3 *Solanum torvum* Sw. (Solanaceae)		P14 *Praxelis clematidae* RM King & H. Rob (Asteraceae)	
	P4 *Mangifera indica* L. (Anacardiaceae)		P15 *Calotropis gigantea* (L.) Dryand. (Apocynaceae)	
	P5 *Dimocarpus longan* Lour. (Sapindaceae)		P16 *Syzygium antisepticum* (Blume) Merr. & L. M. Perry (Myrtaceae)	
	P6 *Sesbania grandiflora* (L.) Poir. (Fabaceae)		P17 *Solanum incanum* L. (Solanaceae)	
	P7 *Sandoricum koetjape* (Burm. f.) Merr. (Meliaceae)		P18 *Bouae burmanica* Griff. (Anacardiaceae)	
	P8 *Zea mays* L. (Poaceae)		P19 *Litchi chinensis* Sonn. (Sapindaceae)	
	P9 *Tridax procumbens* (L.) L. (Asteraceae)		P20 *Carica papaya* L. (Caricaceae)	
	P10 *Gomphrena globosa* L. (Amaranthaceae)		P21 *Rhinacanthus nasutus* (L.) Kurz. (Acanthaceae)	
	P11 *Capsicum annuum* L. (Solanaceae)		P22 *Citrus aurantifolia* (Christm.) Swingle (Rutaceae)	
**Network**	**Plant species**			

P23 *Nicotiana tabacum* L. (Solanaceae)	P37 *Solanum lycopersicum* L. (Solanaceae)
P24 *Telosma cordata* (Burm. f.) Merr. (Apocynaceae)	P38 *Coffea arabica* L. (Rubiaceae)
P25 *Ixora chinensis* Lamk. (Rubiaceae)	P39 *Muntingia calabura* L. (Muntingiaceae)
P26 *Ocimum basilicum* L. (Lamiaceae)	P40 *Cocos nucifera* L. (Arecaceae)
P27 *Ocimum tenuiflorum* L. (Lamiaceae)	P41 *Moringa oleifera* Lam. (Moringaceae)
P28 *Coccinia grandis* (L.) Voigt (Cucurbitaceae)	P42 *Justicia gendarussa* Burm. f. (Acanthaceae)
P29 *Centrosema pubescens* Benth. (Fabaceae)	P43 *Tagetes erecta* L. (Asteraceae)
P30 *Tecoma stans* (L.) Kunth (Bignoniaceae)	P44 *Streblus asper* Lour. (Moraceae)
P31 *Asystasia gangetica* (L.) T. Anderson subsp. Gangetica (Acanthaceae)	P45 *Glossocardia bidens* (Retz.) Veldkamp (Asteraceae)
P32 *Benincasa hispida* (Thunb.) Cogn. (Cucurbitaceae)	P46 *Hygrophila erecta* (Burm. f.) Hochr. (Acanthaceae)
P33 *Carissa carandas* L. (Apocynaceae)	P47 *Zinnia violacea* Cav. (Asteraceae)
P34 *Brassica juncea* (L.) Czern. (Brassicaceae)	P48 *Bidens pilosa* L. (Asteraceae)
P35 *Eryngium foetidum* L. (Apiaceae)	P49 *Areca catechu* L. (Arecaceae)
P36 *Anethum graveolens* L. (Apiaceae)	

Pollinator species

A1 *Apis cerana*	A23 *Amegilla sp.3*	A45 *Formicid sp.3*	A67 *Dolichopodid sp.2*
A2 *Apis florea*	A24 *Megachile sp.2*	A46 *Formicid sp.11*	A68 *Musca sp.*
A3 *Amegilla sp.1*	A25 *Apidae sp.2*	A47 *Formicid sp.13*	A69 *Syritta sp.*
A4 *Amegilla sp.2*	A26 *Xylocopa sp.3*	A48 Banded fly	A70 *Tephritidae sp.*
A5 *Xylocopa sp.1*	A27 *Apidae sp.4*	A49 Small-banded fly	A71 *Helophilu sp.*
A6 *Xylocopa aestuans*	A28 *Vespid sp.5*	A50 *Sarcophagidae sp.5*	A72 *Mesembrius bengalensis*
A7 *Halictid sp.1*	A29 *Vespid sp.6*	A51 *Sarcophagidae sp.6*	A73 *Episyrphus sp.2*
A8 *Tetragonilla sp.2*	A30 *Vespid sp.8*	A52 *Sarcophagidae sp.7*	A74 *Stomorhina sp.*
A9 *Tetragonilla sp.3*	A31 *Vespid sp.9*	A53 *Sarcophagidae sp.4*	A75 *Stratiomyid sp.7*
A10 *Tetragonilla sp.4*	A32 *Vespid sp.1*	A54 *Calliphoridae sp.1*	A76 *Episyrphus sp.1*
A11 *Tetragonilla sp.5*	A33 *Megascolia sp.1*	A55 *Helophilu sp.*	A77 *Lixophaga sp.*
A12 *Halictid sp.2*	A34 *Megascolia sp.5*	A56 *Stratiomyid sp.9*	A78 *Stratiomyid sp.3*
A13 *Andrena sp.2*	A35 *Eumeninae sp.1*	A57 *Stratiomyid sp.6*	A79 Black-white fly
A14 *Andrena sp.3*	A36 *Vespid sp.4*	A58 *Lucilia illustris*	A80 *Stratiomyid sp.4*
A15 *Halictid sp.3*	A37 *Eumeninae sp.2*	A59 *Sarcophaga sp.*	A81 *Drosophila sp.2*
A16 *Ceratina sp.*	A38 *Polistinae sp.1*	A60 *Sarcophagidae sp.3*	A82 *Drosophila sp.1*
A17 *Symmorphus sp.*	A39 *Vespid sp.1*	A61 *Calliphoridae sp.2*	A83 *Eurema hecabe*
A18 *Apidae sp.5*	A40 *Vespid sp.2*	A62 *Muscid sp.1*	A84 *Papilio palytes*
A19 *Halictid sp.7*	A41 *Eumeninae sp.3*	A63 *Muscid sp.2*	A85 *Castalius rosimon*
A20 *Andrena sp.1*	A42 *Vespid sp.3*	A64 *Muscid sp.3*	A86 *Neptis hylas*
A21 *Halictid sp.6*	A43 *Formicid sp.4*	A65 *Muscid sp.4*	A87 *Papilio memnon*
A22 *Halictid sp.4*	A44 *Formicid sp.5*	A66 *Tephritidae sp.1*	A88 *Catopsilia pomona*

**Network**	**Pollinator species**

A89 *Catopsilia scylla*	A95 *Elymnias patna*	A101 *Mordellistina sp.1*	A107 *Glycyphana sp.1*
A90 *Zizina otis*	A96 *Euploea core*	A102 *Glycyphana sp.2*	A108 *Coccinella sp.2*
A91 *Papilio demoleus*	A97 *Acraea sp.*	A103 *Cheilomenes sexmaculata*	A109 *Pentatomidae sp.1*
A92 *Pseudozizeeria maha*	A98 *Elymnias malelas*	A104 *Coccinella sp.3*	A110 *Pentatomidae sp.2*
A93 *Everes huegelii*	A99 *Acraea violae*	A105 *Coccinella trasversalis*	A111 *Spilostethus sp.*
A94 *Danaus chrysippus*	A100 *Coccinella sp.1*	A106 *Coreidae sp.*	A112 *Pentatomidae sp.3*
			A113 Dragonfly (Libellulidae)

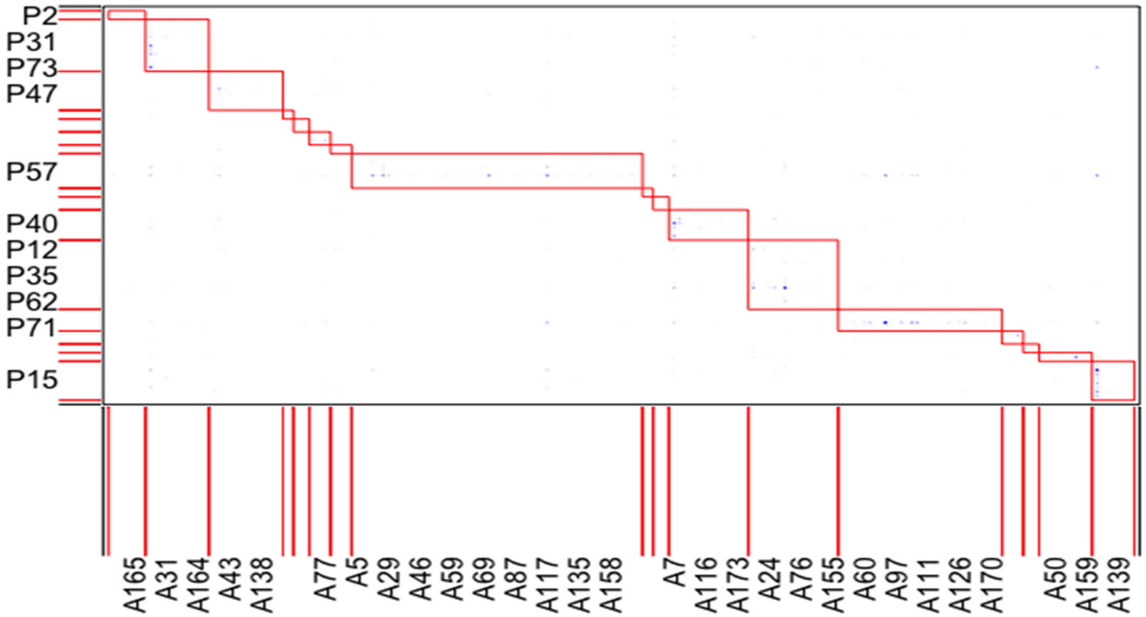

Figure S1. Modularity plot for the network of all study sites.

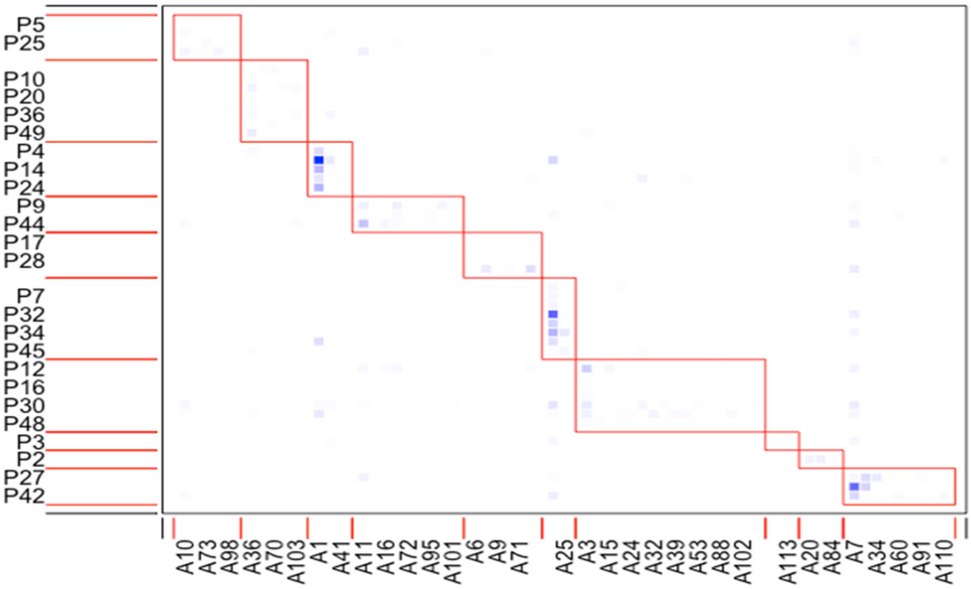

Figure S2. Modularity plot for the invaded network.

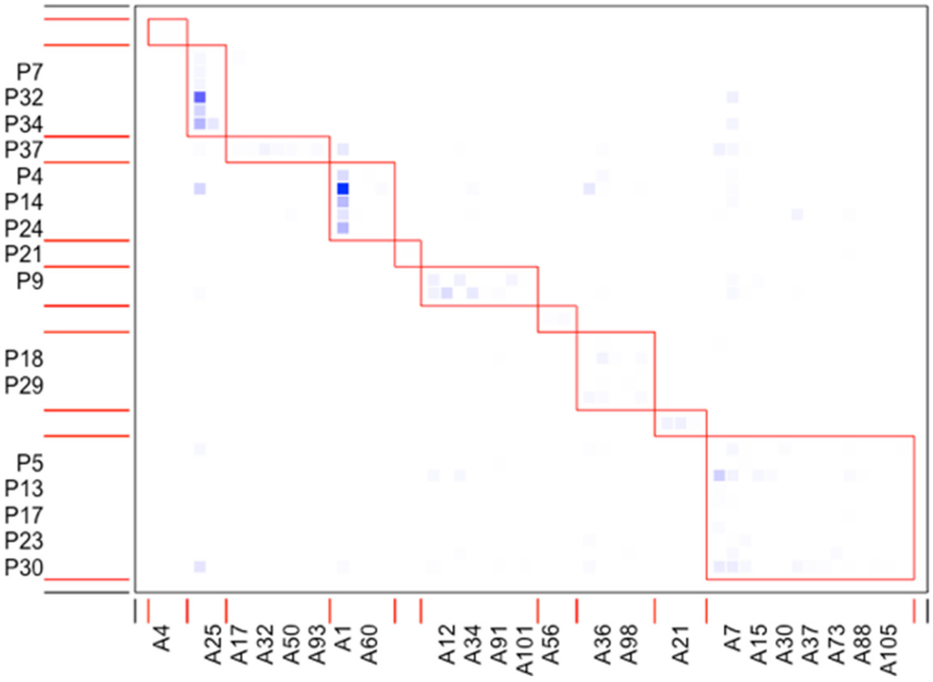

Figure S3. Modularity plot for the non-invaded network.

Table S2. Mean value of participation coefficients (c) and within-module degree (z) values for different pollinator groups. To assess different between invasion states, we used Wilcoxon rank sum test. ^{NS} $p > 0.05$

Order	Mean c-value		p-value	Mean z-value		p-value
	Invaded	Non-invaded		Invaded	Non-invaded	
Hymenoptera	0.23	0.15	0.20 ^{NS}	0.36	0.26	0.75 ^{NS}
Diptera	0.08	0.06	0.61 ^{NS}	-0.26	-0.39	0.63 ^{NS}
Lepidoptera	0.09	0.11	0.63 ^{NS}	-0.32	-0.45	0.61 ^{NS}
Others	0.08	0.00	0.42 ^{NS}	-0.40	-0.52	0.71 ^{NS}

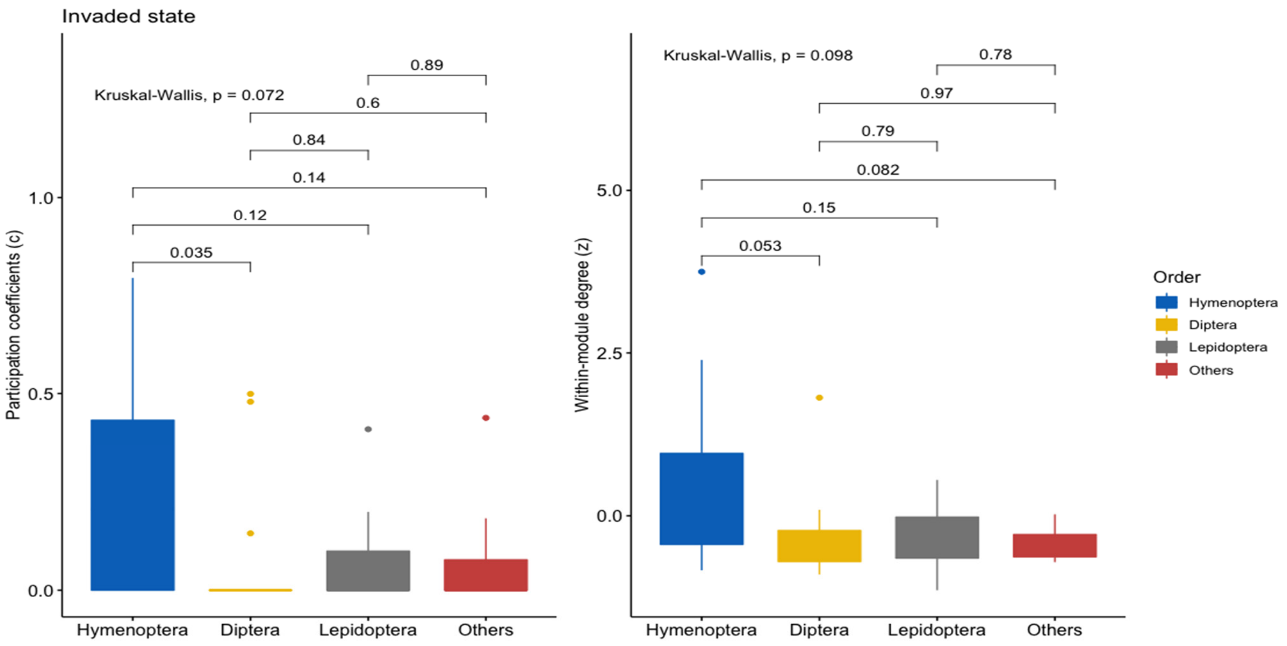

Figure S4. Boxplot showing the participation coefficients (c, left) and the within-module degree (z, right) values for different pollinator groups in the invaded network. To assess different between pollinator groups, we used Wilcoxon rank sum test and used Kruskal-Wallis test assessed different of all pollinator groups.

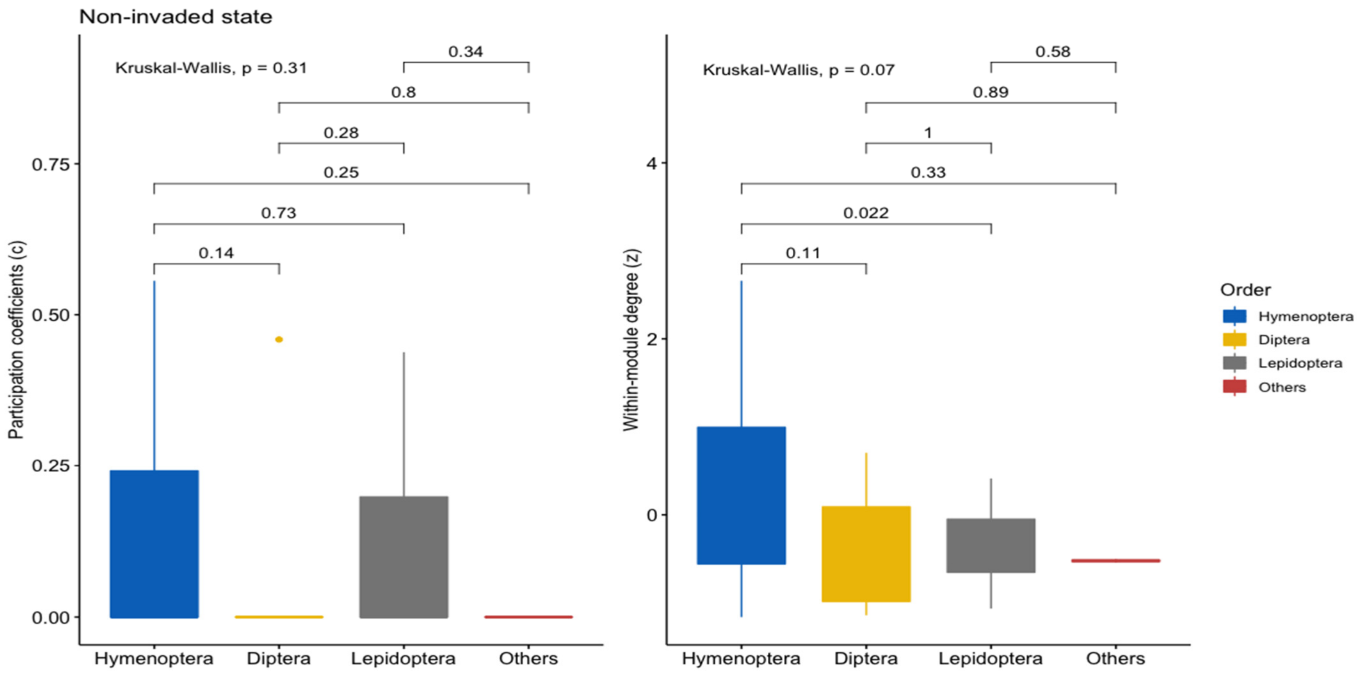

Figure S5. Boxplot showing the participation coefficients (c, left) and the within-module degree (z, right) values for different pollinator groups in the non-invaded network. To assess different between pollinator groups, we used Wilcoxon rank sum test used Kruskal-Wallis test assessed different of all pollinator groups.

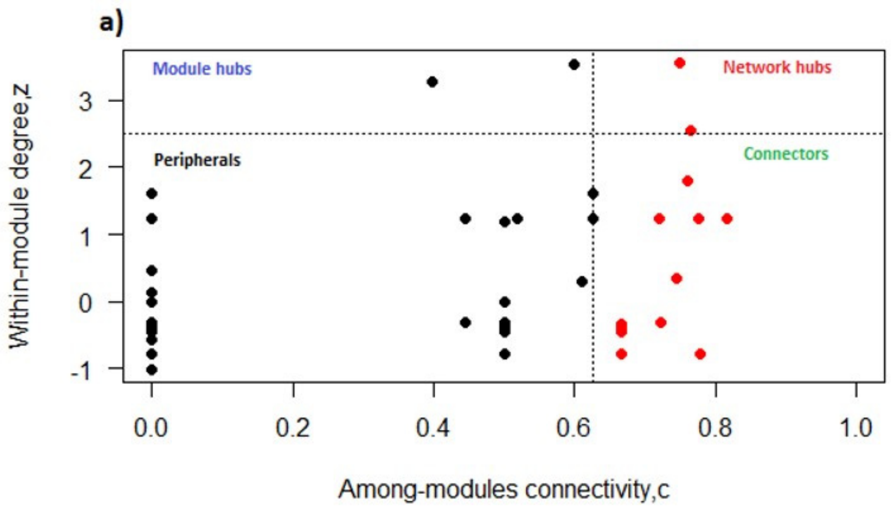

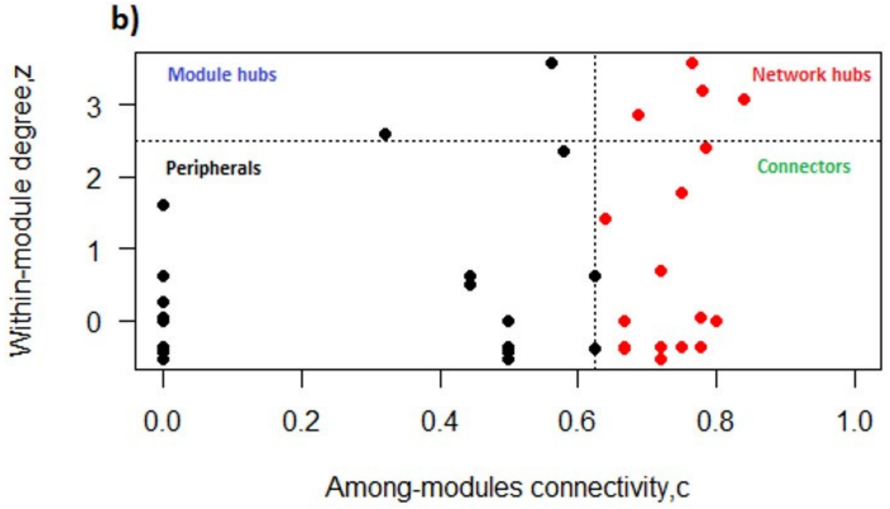

Figure S6. *cz*-plot showing the distribution of pollinators according to their network role of **a**) invaded network, with three network hubs and two module hubs, and **b**) non-invaded network, with four network hubs and two module hubs. Dashed black lines indicate critical values ($c = 0.625$ and $z = 2.5$) according to Olesen et al. (2007).

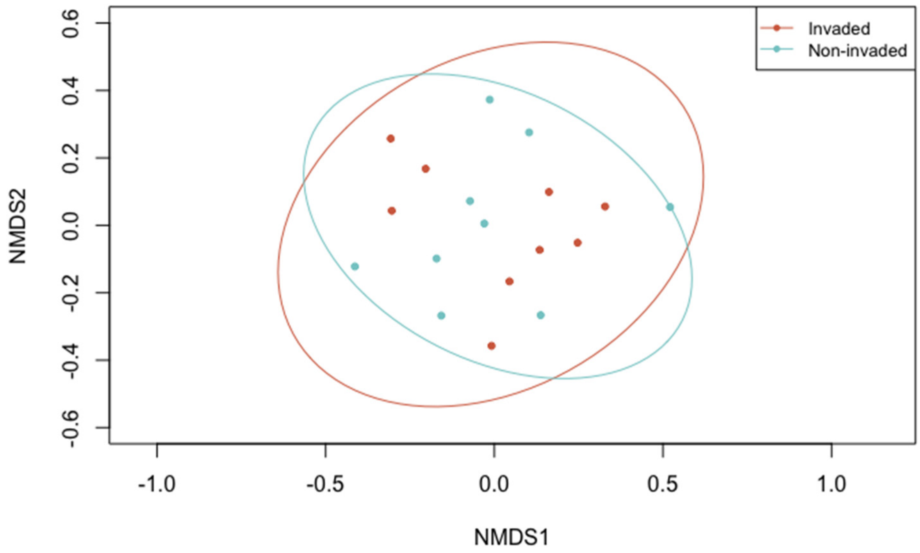

Figure S7. Community composition of all pollinator species at the invaded and non-invaded sites (NMDS plot including 95% ellipses; Stress = 0.17).

Table S3. Generalized Linear Models (GLMs) selection for each dependent variable.

Dependent variables	Model	AICc
**All pollinator richness** (Agriculture 3km)	All richness ~ A3km*P.richness+system	127.57
	All richness ~ A3km*P.richness*flower+system	128.94
	All richness ~ A3km *P.richness+flower+system	129.42
	All richness ~ A3km *P.richness*system	132.17
	All richness ~ A3km *P.richness*flower*system	135.19
	All richness ~ A3km +system	135.99
	All richness ~ A3km +P.richness+system	136.73
	All richness ~ A3km *system	137.17
	All richness ~ A3km+P.richness+flower+system	137.45
**All pollinator richness** (Forest 3km)	All richness ~ F3km*P.richness*flower*system	124.55
	All richness ~ F3km*P.richness*system	128.97
	All richness ~ F3km*P.richness*flower+system	131.05
	All richness ~ F3km*P.richness+system	135.46
	All richness ~ F3km+system	137.28
	All richness ~ F3km*P.richness+flower+system	137.34
	All richness ~ F3km*system	138.04
	All richness ~ F3km+P.richness+system	138.47
	All richness ~ F3km+P.richness+flower+system	139.07
**All pollinator richness** (Urban 3km)	All richness ~ U3km*P.richness*flower*system	124.24
	All richness ~ U3km*P.richness+flower+system	129.36
	All richness ~ U3km+P.richness+system	129.98
	All richness ~ U3km*P.richness*system	131.86
	All richness ~ U3km*P.richness*flower+system	133.36
	All richness ~ U3km+system	137.03
	All richness ~ U3km*system	137.06
	All richness ~ U3km+P.richness+system	138.43
	All richness ~ U3km+P.richness+flower+system	139.27
	Hymenoptera ~ A3km*P.richness+system	111.61

Dependent variables	Model	AICc
**Hymenoptera richness** (Agriculture 3km)	Hymenoptera ~ A3km*P.richness*flower+system	112.52
	Hymenoptera ~ A3km*P.richness+flower+system	113.33
	Hymenoptera ~ A3km*P.richness*flower*system	115.23
	Hymenoptera ~ A3km*P.richness*system	116.69
	Hymenoptera ~ A3km+system	119.10
	Hymenoptera ~ A3km*system	121.03
	Hymenoptera ~ A3km+P.richness+system	121.10
	Hymenoptera ~ A3km+P.richness+flower+system	123.05
**Hymenoptera richness** (Forest 3km)	Hymenoptera ~ F3km*P.richness*flower*system	112.10
	Hymenoptera ~ F3km*P.richness*system	113.33
	Hymenoptera ~ F3km+system	120.29
	Hymenoptera ~ F3km*P.richness+system	120.35
	Hymenoptera ~ F3km+P.richness+system	122.16
	Hymenoptera ~ F3km*P.richness+flower+system	122.212
	Hymenoptera ~ F3km*system	122.215
	Hymenoptera ~ F3km*P.richness*flower+system	122.89
	Hymenoptera ~ F3km+P.richness+flower+system	123.97
**Hymenoptera richness** (Urban 3km)	Hymenoptera ~ U3km*P.richness*flower*system	111.63
	Hymenoptera ~ U3km*P.richness+system	117.14
	Hymenoptera ~ U3km*P.richness*system	118.17
	Hymenoptera ~ U3km*P.richness+flower+system	118.76
	Hymenoptera ~ U3km*system	119.98
	Hymenoptera ~ U3km+system	120.10
	Hymenoptera ~ U3km*P.richness*flower+system	121.37
	Hymenoptera ~ U3km+P.richness+system	122.00
	Hymenoptera ~ U3km+P.richness+flower+system	123.97
**Diptera richness** (Agriculture 3km)	Diptera ~ A3km+system	93.58
	Diptera ~ A3km*system	93.99
	Diptera ~ A3km+P.richness+system	95.24

Dependent variables	Model	AICc
	Diptera ~ A3km*P.richness+system	95.33
	Diptera ~ A3km*P.richness+flower+system	97.22
	Diptera ~ A3km+P.richness+flower+system	97.24
	Diptera ~ A3km*P.richness*flower+system	98.56
	Diptera ~ A3km*P.richness*system	98.70
	Diptera ~ A3km*P.richness*flower*system	108.20
**Diptera richness** (Forest 3km)	Diptera ~ F3km*system	93.22
	Diptera ~ F3km+system	93.55
	Diptera ~ F3km+P.richness+system	95.22
	Diptera ~ F3km*P.richness+system	95.69
	Diptera ~ F3km*P.richness*flower+system	95.90
	Diptera ~ F3km+P.richness+flower+system	97.22
	Diptera ~ F3km*P.richness+flower+system	97.41
	Diptera ~ F3km*P.richness*system	98.78
	Diptera ~ F3km*P.richness*flower*system	105.53
**Diptera richness** (Urban 3km)	Diptera ~ U3km+system	93.24
	Diptera ~ U3km*P.richness+system	93.70
	Diptera ~ U3km+P.richness+system	94.94
	Diptera ~ U3km*system	95.19
	Diptera ~ U3km*P.richness+flower+system	95.69
	Diptera ~ U3km+P.richness+flower+system	96.86
	Diptera ~ U3km*P.richness*system	99.00
	Diptera ~ U3km*P.richness*flower+system	99.01
	Diptera ~ U3km*P.richness*flower*system	105.15
**Lepidoptera richness** (Agriculture 3km)	Lepidoptera ~ A3km+P.richness+flower+system	76.31
	Lepidoptera ~ A3km+system	77.48
	Lepidoptera ~ A3km+P.richness+system	77.92
	Lepidoptera ~ A3km*P.richness+flower+system	78.17
	Lepidoptera ~ A3km*system	79.26

Dependent variables	Model	AICc
	Lepidoptera ~ A3km*P.richness+system	79.91
	Lepidoptera ~ A3km*P.richness*flower+system	82.00
	Lepidoptera ~ A3km*P.richness*system	84.25
	Lepidoptera ~ A3km*P.richness*flower*system	93.12
**Lepidoptera richness** (Forest 3km)	Lepidoptera ~ F3km+P.richness+system	76.28
	Lepidoptera ~ F3km+P.richness+flower+system	76.35
	Lepidoptera ~ F3km+system	76.39
	Lepidoptera ~ F3km*P.richness+flower+system	77.52
	Lepidoptera ~ F3km*P.richness+system	78.06
	Lepidoptera ~ F3km*system	78.31
	Lepidoptera ~ F3km*P.richness*system	79.57
	Lepidoptera ~ F3km*P.richness*flower+system	80.71
	Lepidoptera ~ F3km*P.richness*flower*system	82.63
**Lepidoptera richness** (Urban 3km)	Lepidoptera ~ U3km+P.richness+flower+system	79.09
	Lepidoptera ~ U3km+system	79.94
	Lepidoptera ~ U3km*P.richness+flower+system	80.55
	Lepidoptera ~ U3km+P.richness+system	81.26
	Lepidoptera ~ U3km*system	81.49
	Lepidoptera ~ U3km*P.richness+system	83.22
	Lepidoptera ~ U3km*P.richness*flower+system	83.86
	Lepidoptera ~ U3km*P.richness*flower*system	83.94
	Lepidoptera ~ U3km*P.richness*system	87.97
**All pollinator visitation rate** (Agriculture 3km)	AVR ~ A3km+system	160.20
	AVR ~ A3km*P.richness*system	160.81
	AVR ~ A3km*system	161.54
	AVR ~ A3km+P.richness+system	161.94
	AVR ~ A3km*P.richness+system	163.35
	AVR ~ A3km+P.richness+flower+system	163.65
	AVR ~ A3km*P.richness+flower+system	164.71

Dependent variables	Model	AICc
	AVR ~ A3km*P.richness*flower*system	168.39
	AVR ~ A3km*P.richness*flower+system	169.80
**All pollinator visitation rate** (Forest 3km)	AVR ~ F3km*P.richness*flower*system	126.97
	AVR ~ F3km+system	160.94
	AVR ~ F3km+P.richness+system	162.54
	AVR ~ F3km*system	162.63
	AVR ~ F3km+P.richness+flower+system	164.25
	AVR ~ F3km*P.richness+system	164.54
	AVR ~ F3km*P.richness+flower+system	166.18
	AVR ~ F3km*P.richness*flower+system	169.99
	AVR ~ F3km*P.richness*system	170.01
**All pollinator visitation rate** (Urban 3km)	AVR ~ U3km*system	160.45
	AVR ~ U3km+system	161.30
	AVR ~ U3km*P.richness+system	162.03
	AVR ~ U3km+P.richness+system	162.67
	AVR ~ U3km*P.richness+flower+system	164.03
	AVR ~ U3km+P.richness+flower+system	164.64
	AVR ~ U3km*P.richness*system	164.87
	AVR ~ U3km*P.richness*flower*system	167.09
	AVR ~ U3km*P.richness*flower+system	167.59
**Hymenoptera visitation rate** (Agriculture 3km)	HVR~ A3km+system	157.96
	HVR ~ A3km*system	158.19
	HVR ~ A3km+P.richness+system	159.79
	HVR ~ A3km*P.richness*system	161.10
	HVR ~ A3km*P.richness+system	161.44
	HVR ~ A3km+P.richness+flower+system	161.46
	HVR ~ A3km*P.richness*flower*system	163.24
	HVR ~ A3km*P.richness+flower+system	163.25
	HVR ~ A3km*P.richness*flower+system	166.59

Dependent variables	Model	AICc
**Hymenoptera visitation rate** (Forest 3km)	HVR ~ F3km*P.richness*flower*system	120.13
	HVR ~ F3km+syste	158.96
	HVR ~ F3km+P.richness+system	160.92
	HVR ~ F3km*system	160.94
	HVR ~ F3km+P.richness+flower+system	162.52
	HVR ~ F3km*P.richness+system	162.90
	HVR ~ F3km*P.richness+flower+system	164.33
	HVR ~ F3km*P.richness*flower+system	166.75
	HVR ~ F3km*P.richness*system	168.36
**Hymenoptera visitation rate** (Urban 3km)	HVR ~ U3km*P.richness*flower*system	155.01
	HVR ~ U3km*system	157.86
	HVR ~ U3km+system	158.79
	HVR ~ U3km*P.richness+flower+system	160.24
	HVR ~ U3km*P.richness*flower+syste	160.33
	HVR ~ U3km*P.richness+system	160.77
	HVR ~ U3km+P.richness+system	160.79
	HVR ~ U3km+P.richness+flower+system	161.11
	HVR ~ U3km*P.richness*system	162.95
**Diptera visitation rate** (Agriculture 3km)	DVR ~ A3km*P.richness*system	134.27
	DVR ~ A3km*P.richness*flower*system	137.61
	DVR ~ A3km+P.richness+system	139.98
	DVR ~ A3km+system	140.49
	DVR ~ A3km*P.richness+system	140.53
	DVR ~ A3km+P.richness+flower+syste	141.90
	DVR ~ A3km*P.richness+flower+system	142.10
	DVR ~ A3km*system	142.10
	DVR ~ A3km*P.richness*flower+system	145.24
**Diptera visitation rate** (Forest 3km)	DVR ~ F3km*P.richness*flower*system	81.85
	DVR ~ F3km*P.richness*flower+system	137.53

Dependent variables	Model	AICc
	DVR ~ F3km*system	139.22
	DVR ~ F3km+system	140.64
	DVR ~ F3km+P.richness+system	140.70
	DVR ~ F3km*P.richness+system	142.34
	DVR ~ F3km+P.richness+flower+system	142.54
	DVR ~ F3km*P.richness+flower+system	143.83
	DVR ~ F3km*P.richness*system	144.85
**Diptera visitation rate** (Urban 3km)	DVR ~ U3km*P.richness+system	135.64
	DVR ~ U3km*P.richness+flower+system	137.00
	DVR ~ U3km+system	140.04
	DVR ~ U3km+P.richness+system	140.15
	DVR ~ U3km*P.richness*system	140.60
	DVR ~ U3km*P.richness*flower+system	141.04
	DVR ~ U3km+P.richness+flower+system	141.21
	DVR ~ U3km*system	141.63
	DVR ~ U3km*P.richness*flower*system	143.23
**Lepidoptera visitation rate** (Agriculture 3km)	LVR ~ A3km*P.richness*flower*system	56.57
	LVR ~ A3km*P.richness*flower+system	147.85
	LVR ~ A3km*P.richness*system	156.24
	LVR ~ A3km+system	158.51
	LVR ~ A3km+P.richness+flower+system	159.50
	LVR ~ A3km*system	159.67
	LVR ~ A3km+P.richness+system	160.24
	LVR ~ A3km*P.richness+flower+system	160.55
	LVR ~ A3km*P.richness+system	162.06
**Lepidoptera visitation rate** (Forest 3km)	LVR ~ F3km*P.richness*flower*system	91.54
	LVR ~ F3km*P.richness*system	150.27
	LVR ~ F3km*P.richness+flower+system	157.03
	LVR ~ F3km+system	158.96

Dependent variables	Model	AICc
	LVR ~ F3km+P.richness+flower+system	159.59
	LVR ~ F3km*P.richness*flower+system	159.62
	LVR ~ F3km*system	160.29
	LVR ~ F3km+P.richness+system	160.59
	LVR ~ F3km*P.richness+system	161.57
**Lepidoptera visitation rate** (Urban 3km)	LVR ~ U3km*P.richness*flower*system	135.57
	LVR ~ U3km+system	159.63
	LVR ~ U3km*P.richness+flower+system	159.73
	LVR ~ U3km*P.richness+system	160.50
	LVR ~ U3km+P.richness+system	160.96
	LVR ~ U3km+P.richness+flower+system	161.20
	LVR ~ U3km*system	161.32
	LVR ~ U3km*P.richness*flower+system	162.70
	LVR ~ U3km*P.richness*system	163.21

Table S4. Results of generalized Linear Models (GLMs) best fitted for each dependent variable.

Dependent variables	Explanatory fixed variable	Estimate	SE	z-value	P-value
	Intercept	-1.556	1.493	-1.042	0.297
	A3km	0.070	0.020	3.498	0.0005***
**All pollinator richness** (Agriculture 3km)	Plant richness	0.157	0.051	3.087	0.002**
	system	0.013	0.094	0.142	0.887
	A3km:Plant richness	-0.002	0.0007	-3.327	0.0009***
	Intercept	-1.612	2.853	-0.565	0.572
	F3km	1.275	-0.374	3.406	0.0007***
	Plant richness	-0.183	-0.103	1.789	0.074
	flower	2.318e-03	1.262e-03	1.837	0.066
	system	9.903	4.718	2.099	0.036*
	F3km:Plant richness	-4.886e-02	1.496e-02	-3.265	0.001**
	F3km:flower	-5.143e-04	1.612e-04	-3.190	0.001**
**All pollinator richness** (Forest 3km)	Plant richness:flower	-8.361e-05	4.529e-05	-1.846	0.065
	F3km:system	-1.540	-0.696	-2.215	0.028*
	Plant richness:system	-0.340	-0.181	-1.884	0.060
	flower:system	-4.346e-03	1.975e-03	-2.201	0.028*
	F3km:Plant richness:flower	1.928e-05	6.275e-06	3.073	0.002**
	F3km:Plant richness:system	5.661e-02	2.929e-02	1.933	0.054
	F3km:flower:system	6.138e-04	2.731e-04	2.248	0.025*
	Plant richness:flower:system	1.492e-04	7.389e-05	2.019	0.043*
	F3km:Plant richness:flower:system	-2.220e-05	1.124e-05	-1.975	0.048*
	Intercept	35.30	9.478	3.725	0.0002***
	U3km	-2.519	0.704	-3.580	0.0003***
**All pollinator richness** (Urban 3km)	Plant richness	-1.081	0.342	-3.166	0.002**
	flower	-1.478e-02	4.594e-03	-3.217	0.001**
	system	-102.8	40.454	-2.309	0.02*
	U3km:Plant richness	8.569e-02	2.537e-02	3.378	0.0007***

	U3km:flower	1.106e-03	3.379e-04	3.274	0.001**
	Plant richness:flower	4.851e-04	1.560e-04	3.109	0.002**
	U3km:system	10.784	9.455	1.887	0.06
	Plant richness:system	3.470	1.537	2.257	0.024*
	flower:system	2.448e-02	7.464e-03	3.280	0.001**
	U3km:Plant richness:flower	-3.678e-05	1.167e-05	-3.152	0.002**
	U3km:Plant richness:system	-0.650	0.351	-1.849	0.064
	U3km:flower:system	-4.196e-03	1.887e-03	-2.224	0.026*
	Plant richness:flower:system	-7.431e-04	2.285e-04	-3.252	0.001**
	U3km:Plant richness:flower:system	1.475e-04	6.864e-05	2.149	0.032*
**Hymenoptera richness** (Agriculture 3km)	Intercept	-4.744	2.211	-2.146	0.03*
	A3km	0.099	0.029	3.371	0.0007***
	Plant richness	0.241	0.073	3.307	0.0009***
	system	-0.024	0.134	-0.177	0.859
	A3km:Plant richness	-0.003	0.0009	-3.332	0.0009***
**Hymenoptera richness** (Forest 3km)	Intercept	-5.305	3.849	-1.378	0.168
	F3km	1.027	-0.502	2.044	0.041*
	Plant richness	-0.289	-0.137	2.105	0.035*
	flower	3.242e-03	1.686e-03	1.923	0.054
	system	20.176	6.738	3.229	0.001**
	F3km:Plant richness	-3.682e-02	1.978e-02	-1.862	0.063
	F3km:flower	-3.984e-04	2.228e-04	-1.788	0.074
	Plant richness:flower	-1.172e-04	6.038e-05	-1.941	0.052
	F3km:system	-2.393	0.971	-2.465	0.014*
	Plant richness:system	-0.762	-0.255	-2.983	0.003**
	flower:system	-8.74e-03	2.801e-03	-3.120	0.002**
	F3km:Plant richness:flower	1.403e-05	8.591e-06	1.633	0.103
	F3km:Plant richness:system	8.853e-02	4.062e-02	2.179	0.029*
	F3km:flower:system	8.671e-04	3.791e-04	2.179	0.022*

	Plant richness:flower:system	3.082e-04	1.043e-04	2.945	0.003*
	F3km:Plant richness:flower:system	-3.209e-05	1.557e-05	-2.061	0.039*
**Hymenoptera richness** (Urban 3km)	Intercept	20.067	10.158	1.785	0.074
	U3km	-1.864	0.876	-2.129	0.033*
	Plant richness	-0.602	0.417	-1.443	0.149
	flower	-7.652e-03	5.668e-03	-1.350	0.177
	system	-200.087	60.438	-3.242	0.001**
	U3km:Plant richness	6.328e-02	3.155e-02	2.005	0.045*
	U3km:flower	7.309e-04	4.221e-04	1.732	0.083
	Plant richness:flower	2.497e-04	1.911e-04	1.307	0.191
	U3km:system	40.309	10.371	3.143	0.002**
	Plant richness:system	7.097	2.217	3.202	0.001**
	flower:system	3.23e-02	1.022e-02	3.160	0.002**
	U3km:Plant richness:flower	-2.46e-05	1.454e-05	-1.692	0.091
	U3km:Plant richness:system	-1.589	-0.509	-3.120	0.002**
	U3km:flower:system	-8.82e-03	2.721e-03	-3.242	0.001**
	Plant richness:flower:system	-9.178e-04	3.068e-04	-2.991	0.003**
	U3km:Plant richness:flower:system	3.175e-04	9.893e-05	3.209	0.001**
**Diptera richness** (Agriculture 3km)	Intercept	2.105	0.497	4.237	2.27e-05 ***
	A3km	0.001	0.006	0.165	0.869
	system	0.103	0.161	0.639	0.523
**Diptera richness** (Forest 3km)	Intercept	2.301	0.139	16.582	<2e-16 ***
	F3km	-0.017	0.013	-1.281	0.200
	system	-0.129	0.220	-0.584	0.559
	F3km:system	0.026	0.017	1.522	0.128
**Diptera richness** (Urban 3km)	Intercept	2.076	0.211	9.809	<2e-16 ***
	U3km	0.011	0.017	0.612	0.541
	system	0.119	0.159	0.746	0.456

**Lepidoptera richness** (Agriculture 3km)	Intercept	0.159	1.282	0.124	0.902
	A3km	2.253e-02	1.356e-02	1.661	0.097
	Plant richness	-3.33e-02	3.096e-02	-1.076	0.282
	flower	1.416e-04	7.039e-05	2.012	0.044*
	system	-0.258	0.278	-0.928	0.354
**Lepidoptera richness** (Forest 3km)	Intercept	2.943	0.976	3.017	0.003**
	F3km	-0.038	0.017	-2.222	0.026*
	Plant richness	-0.044	0.031	-1.411	0.158
	system	-0.336	0.273	-1.231	0.218
**Lepidoptera richness** (Urban 3km)	Intercept	1.525	0.921	1.657	0.098
	U3km	-1.861e-02	3.483e-02	-0.534	0.593
	Plant richness	1.754e-02	2.964e-02	-0.592	0.554
	flower	1.842e-04	8.832e-05	2.086	0.037*
	system	-0.398	0.276	-1.442	0.149
**All pollinator visitation rate** (Agriculture 3km)	Intercept	9.863	27.480	0.359	0.725
	A3km	0.356	0.343	1.036	0.317
	system	-0.766	8.914	-0.086	0.933
**All pollinator visitation rate** (Forest 3km)	Intercept	-4.904e+02	1.560e+02	-3.144	0.088
	F3km	87.27	18.26	4.779	0.041*
	Plant richness	19.82	5.636	3.516	0.072
	flower	0.250	6.819e-02	3.681	0.067
	system	1.030e+03	245.2	4.199	0.052
	F3km:Plant richness	-3.436	0.707	-4.855	0.039*
	F3km:flower	-4.080e-02	7.970e-03	-5.119	0.036*
	Plant richness:flower	-9.149e-03	2.449e-03	-3.735	0.065
	F3km:system	-100.701	35.26	-4.823	0.04*
	Plant richness:system	-30.973	9.383	-4.234	0.052
	flower:system	-0.482	0.103	-4.683	0.043*
	F3km:Plant richness:flower	1.576e-03	3.044e-04	5.180	0.035*

	F3km:Plant richness:system	6.844	1.480	4.626	0.044*
	F3km:flower:system	7.586e-02	1.391e-02	5.452	0.032*
	Plant richness:flower:system	1.782e-02	3.855e-03	4.623	0.044*
	F3km:Plant richness:flower:system	-2.957e-03	5.709e-04	-5.180	0.035*
**All pollinator visitation rate** (Urban 3km)	Intercept	28.410	13.974	2.033	0.062
	U3km	0.905	1.241	0.730	0.478
	system	22.321	19.131	1.167	0.263
	U3km:system	-2.962	1.912	-1.549	0.144
**Hymenoptera visitation rate** (Agriculture 3km)	Intercept	2.197	25.823	0.085	0.933
	A3km	0.336	0.323	1.041	0.314
	system	0.553	8.377	0.066	0.948
**Hymenoptera visitation rate** **(**Forest 3km)	Intercept	-303.6	129.0	-2.354	0.143
	F3km	74.05	15.10	4.904	0.039*
	Plant richness	11.71	4.660	2.514	0.129
	flower	0.138	5.639e-02	2.450	0.134
	system	673.4	202.8	3.320	0.08
	F3km:Plant richness	-2.862	0.585	-4.891	0.039*
	F3km:flower	-3.289e-02	6.591e-03	-4.990	0.038*
	Plant richness:flower	-4.903e-03	2.025e-03	-2.241	0.137
	F3km:system	-137.4	29.16	-4.713	0.042*
	Plant richness:system	-25.66	7.760	-3.307	0.081
	flower:system	-0.283	8.513e-02	-3.323	0.08
	F3km:Plant richness:flower	1.265e-03	2.517e-04	5.027	0.037*
	F3km:Plant richness:system	5.450	1.223	4.455	0.047*
	F3km:flower:system	6.084e-02	1.151e-02	5.288	0.034*
	Plant richness:flower:system	1.045e-02	3.187e-03	3.277	0.082
	F3km:Plant richness:flower:system	-2.343e-03	4.721e-04	-4.962	0.038*
**Hymenoptera visitation rate** **(**Urban 3km)	Intercept	1.059e+03	8.692e+02	1.218	0.347
	U3km	-80.23	63.83	-1.173	0.362

	Plant richness	-42.13	31.31	-1.346	0.311
	flower	-0.387	0.416	-0.930	0.451
	system	-5.059e+03	5.009e+03	-1.010	0.419
	U3km:Plant richness	3.186	2.460	1.295	0.325
	U3km:flower	2.953e-02	3.202e-02	0.922	0.454
	Plant richness:flower	1.583e-02	1.409e-02	1.123	0.378
	U3km:system	9.353e+02	1.073e+03	0.872	0.475
	Plant richness:system	1.803e+02	1.731e+02	1.042	0.407
	flower:system	1.012	0.784	1.290	0.326
	U3km:Plant richness:flower	-1.176e-03	1.106e-03	-1.063	0.399
	U3km:Plant richness:system	-34.93	39.91	-0.875	0.474
	U3km:flower:system	-0.204	0.213	-0.958	0.439
	Plant richness:flower:system	-3.364e-02	2.361e-02	-1.425	0.290
	U3km:Plant richness:flower:system	7.488e-03	7.763e-03	0.965	0.437
**Diptera visitation rate** (Agriculture 3km)	Intercept	-536.320	178.742	-3.001	0.013*
	A3km	6.245	2.206	2.831	0.018*
	system	20.157	6.471	3.115	0.011*
	A3km:Plant richness	495.717	190.283	2.605	0.026*
	A3km:system	-0.230	0.079	-2.908	0.016*
	Plant richness:system	-17.838	6.869	-2.597	0.027*
	A3km: Plant richness: system	0.202	0.085	2.366	0.04*
**Diptera visitation rate** (Forest 3km)	Intercept	-408.0	43.57	-9.365	0.011*
	F3km	44.79	5.101	8.781	0.013*
	Plant richness	15.74	1.574	9.997	0.0099**
	flower	0.189	1.905e-02	9.940	0.0099**
	system	347.7	68.51	5.076	0.037*
	F3km:Plant richness	-1.755	0.198	-8.876	0.012*
	F3km:flower	-2.164e-02	2.227e-03	-9.719	0.01*
	Plant richness:flower	-6.910e-03	6.843e-04	-10.099	0.0097**

	F3km:system	-31.71	9.850	-3.219	0.084
	Plant richness:system	-13.08	2.621	-4.991	0.038*
	flower:system	-0.179	2.876e-02	-6.225	0.025*
	F3km:Plant richness:flower	8.292e-04	8.503e-05	9.753	0.01*
	F3km:Plant richness:system	1.199	0.413	2.901	0.101
	F3km:flower:system	1.778e-02	3.887e-03	4.574	0.045*
	Plant richness:flower:system	6.430e-03	1.077e-03	5.971	0.027*
	F3km:Plant richness:flower:system	-6.511e-04	1.595e-04	-4.082	0.055
**Diptera visitation rate** (Urban 3km)	Intercept	42.983	25.759	1.669	0.119
	U3km	-7.588	3.375	-2.248	0.043*
	system	-1.204	0.867	-1.388	0.189
	U3km: Plant richness	0.267	0.112	2.378	0.033*
**Lepidoptera visitation rate** (Agriculture 3km)	Intercept	-7.183e+02	2.685e+03	-0.268	0.814
	A3km	6.804	35.26	0.193	0.865
	Plant richness	-7.782	1.028e+02	-0.076	0.947
	flower	0.211	1.053	0.200	0.860
	system	1.016e+03	2.709e+03	0.375	0.744
	A3km: Plant richness	0.184	1.348	0.137	0.904
	A3km: flower	-1.505e-03	1.389e-02	-0.108	0.924
	Plant richness: flower	4.969e-03	4.020e-02	0.124	0.913
	A3km: system	-11.91	35.51	-0.355	0.769
	Plant richness: system	-4.397	1.040e+02	-0.042	0.970
	flower: system	-0.313	1.063	-0.294	0.796
	A3km: Plant richness: flower	-1.038e-04	5.286e-04	-0.196	0.862
	A3km: Plant richness: system	1.654e-02	1.361	0.012	0.991
	A3km: flower: system	3.219e-03	1.401e-02	0.230	0.840
	Plant richness: flower: system	-5.275e-04	4.067e-02	-0.013	0.991
	A3km: Plant richness: flower:system	3.295e-05	5.344e-04	0.062	0.956
	Intercept	289.3	58.28	4.963	0.038*

**Lepidoptera visitation rate** (Forest 3km)	F3km	-0.210	6.824	-3.075	0.092
	Plant richness	-8.075	2.106	-3.834	0.062
	flower	-2.828e-02	2.548e-02	-1.110	0.383
	system	-496.7	91.65	-5.420	0.032
	F3km:Plant richness	0.593	0.265	2.242	0.154
	F3km:flower	4.638e-03	2.979e-03	1.557	0.260
	Plant richness:flower	7.197e-04	9.153e-04	0.786	0.514
	F3km:system	39.98	13.18	3.034	0.094
	Plant richness:system	16.04	3.507	4.573	0.045*
	flower:system	9.653e-02	3.847e-02	2.509	0.129
	F3km:Plant richness:flower	-1.308e-04	1.137e-04	-1.150	0.369
	F3km:Plant richness:system	-1.366	0.553	-2.471	0.132
	F3km:flower:system	-1.160e-02	5.200e-03	-2.231	0.155
	Plant richness:flower:system	-3.299e-03	1.440e-03	-2.290	0.149
	F3km:Plant richness:flower:system	4.132e-04	2.134e-04	1.937	0.192
**Lepidoptera visitation rate** (Urban 3km)	Intercept	574.3	506.5	1.134	0.374
	U3km	-13.98	39.85	-0.351	0.759
	Plant richness	-9.242	1.825	-0.507	0.663
	flower	-0.433	0.243	-1.784	0.216
	system	757.1	2.919e+03	0.259	0.820
	U3km:Plant richness	-0.107	1.434	-0.074	0.947
	U3km:flower	2.147e-02	1.866e-02	1.151	0.369
	Plant richness:flower	9.762e-03	8.214e-03	1.188	0.357
	U3km:system	-2.896e+02	6.253e+02	-0.463	0.689
	Plant richness:system	-35.97	100.9	-0.357	0.756
	flower:system	0.268	0.457	0.587	0.617
	U3km:Plant richness:flower	-4.268e-04	6.446e-04	-0.662	0.576
	U3km:Plant richness:system	11.37	23.26	0.489	0.673
	U3km:flower:system	3.758e-02	0.124	0.303	0.791

	Plant richness:flower:system	-5.454e-03	1.376e-02	-0.396	0.730
	U3km:Plant richness:flower:system	-1.720e-03	4.524e-03	-0.380	0.740

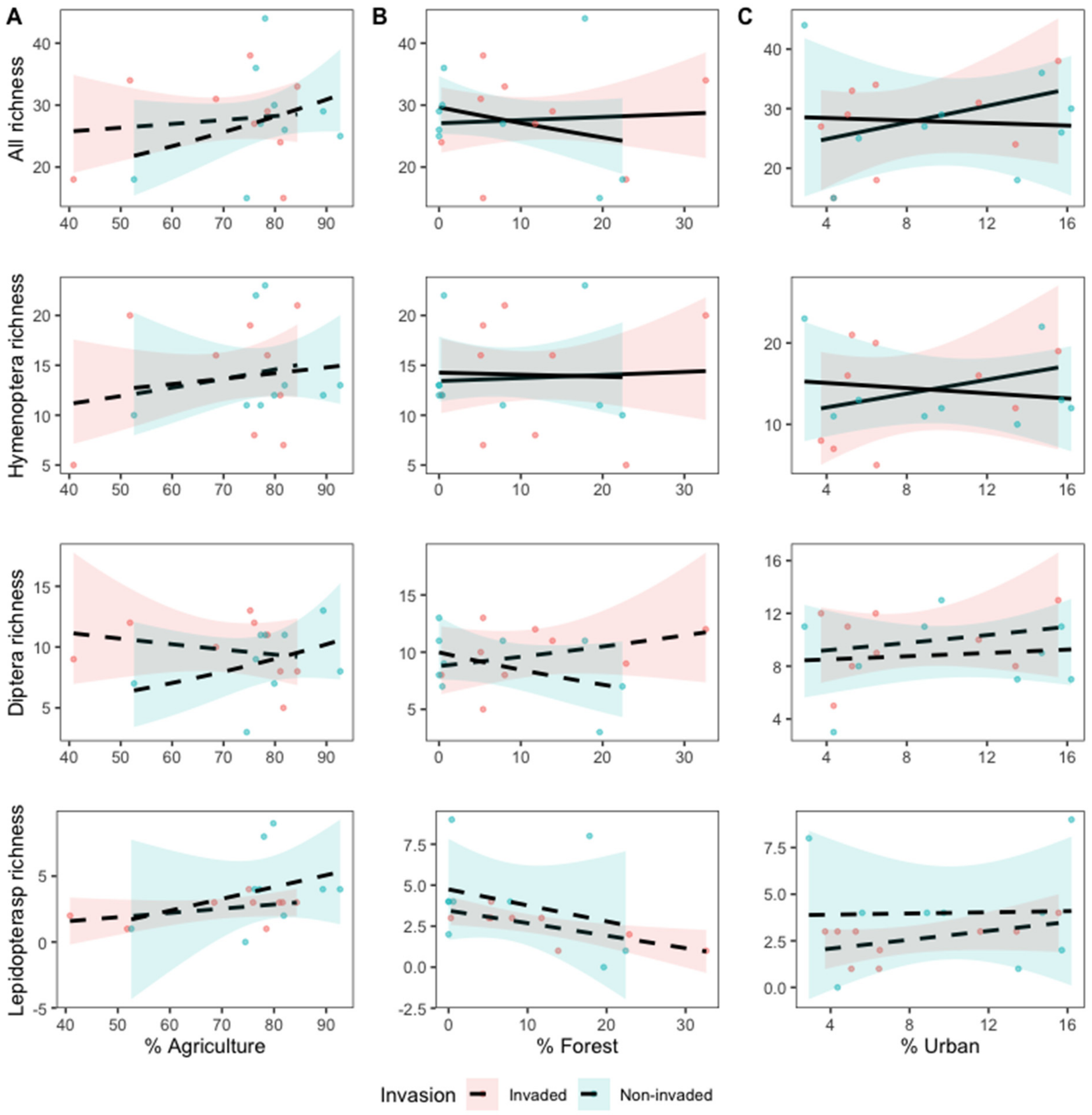

Figure S8. The relationship between invasion state and pollinator richness with relation to the percentage of (A) agriculture, (B) forest, and (C) urban landscape within 3 km radius from each study site. All regressions are plotted with 95% corresponding confident intervals. Solid lines indicate significant associations ($p < 0.05$), whereas dashed lines indicate non-significant relationship ($p > 0.05$).

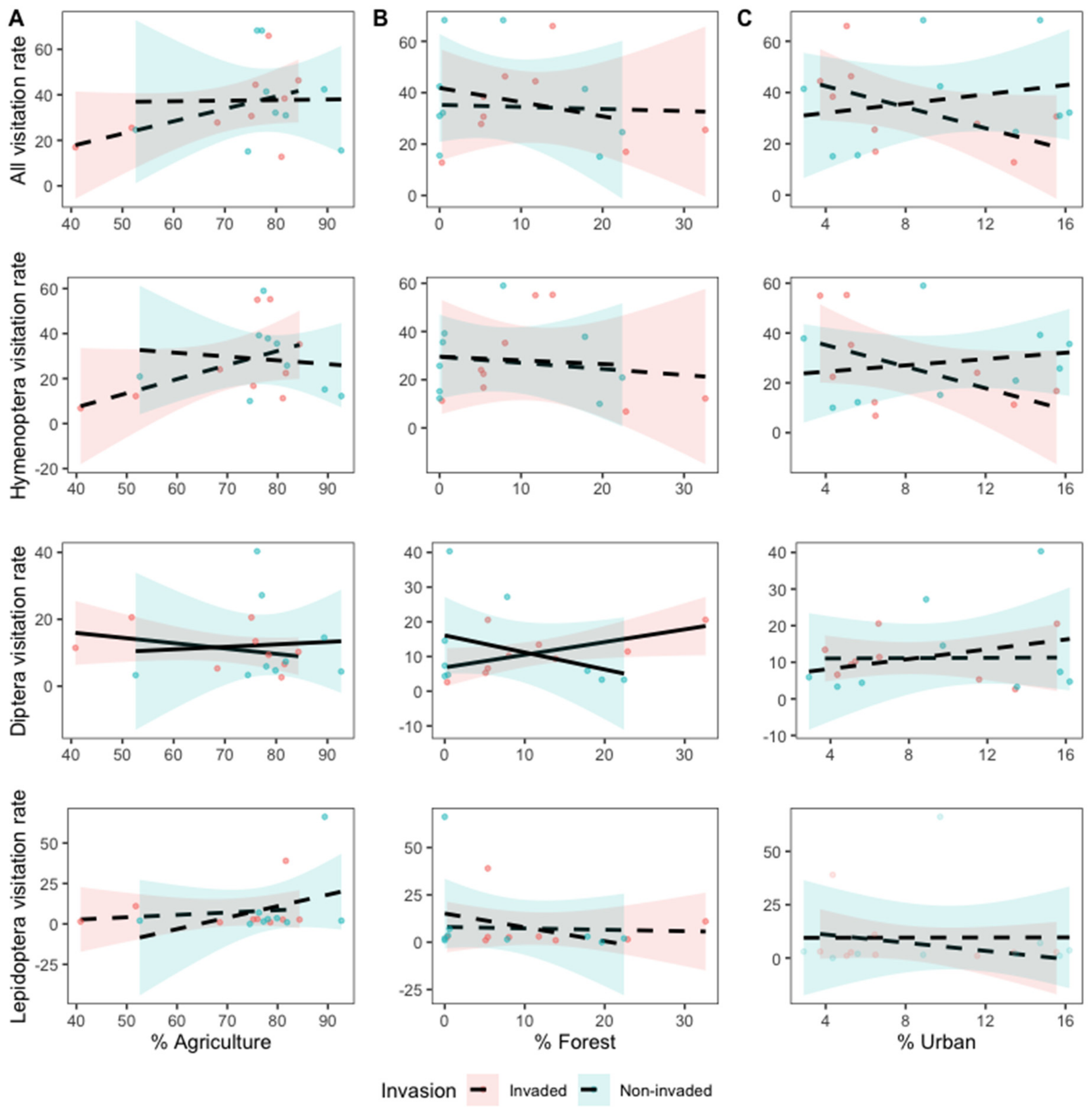

Figure S9. The relationship between invasion state and visitation rates with relation to the percentage of (A) agriculture, (B) forest, and (C) urban landscape within 3 km radius from each study site. All regressions are plotted with 95% corresponding confident intervals. Solid lines indicate significant associations ($p < 0.05$), whereas dashed lines indicate non-significant relationship ($p > 0.05$).
